# An Analytical Model of Leakage Neutron Equivalent Dose for Passively-Scattered Proton Radiotherapy and Validation with Measurements

**DOI:** 10.3390/cancers7020795

**Published:** 2015-05-18

**Authors:** Christopher Schneider, Wayne Newhauser, Jad Farah

**Affiliations:** 1 Department of Physics and Astronomy, Louisiana State University and Agricultural and Mechanical College, 202 Nicholson Hall, Baton Rouge, LA 70803, USA; E-Mail: cschn19@tigers.lsu.edu; 2 Mary Bird Perkins Cancer Center, 4950 Essen Lane, Baton Rouge, LA 70809, USA; 3 Institut de Radioprotection et de Sûreté Nucléaire, Service de Dosimétrie Externe, BP-17, 92262 Fontenay-aux-Roses, France; E-Mail: jad.farah@irsn.fr

**Keywords:** proton therapy, neutron equivalent dose, analytical model

## Abstract

Exposure to stray neutrons increases the risk of second cancer development after proton therapy. Previously reported analytical models of this exposure were difficult to configure and had not been investigated below 100 MeV proton energy. The purposes of this study were to test an analytical model of neutron equivalent dose per therapeutic absorbed dose (H/D) at 75 MeV and to improve the model by reducing the number of configuration parameters and making it continuous in proton energy from 100 to 250 MeV. To develop the analytical model, we used previously published *H/D* values in water from Monte Carlo simulations of a general-purpose beamline for proton energies from 100 to 250 MeV. We also configured and tested the model on in-air neutron equivalent doses measured for a 75 MeV ocular beamline. Predicted *H/D* values from the analytical model and Monte Carlo agreed well from 100 to 250 MeV (10% average difference). Predicted *H/D* values from the analytical model also agreed well with measurements at 75 MeV (15% average difference). The results indicate that analytical models can give fast, reliable calculations of neutron exposure after proton therapy. This ability is absent in treatment planning systems but vital to second cancer risk estimation.

## 1. Introduction

In many cases, proton therapy is dosimetrically advantageous compared to other forms of external beam radiation therapy because it allows for uniform target coverage with lower doses to healthy tissues [1,2,3]. However, proton therapy patients are still exposed to stray radiation, which is not fully understood and not routinely estimated for most patients. Most proton beam treatments are delivered by the passive scattering technique. Stray radiation dose to the patient from passively scattered proton therapy (PSPT) primarily comes from neutrons that leak out of the treatment head [4,5]. This is a concern because neutrons have an enhanced relative biological effectiveness compared with protons [6,7], and even relatively small doses far from the primary treatment field increase the risk of secondary cancers [8]. Commercial treatment planning systems do not take neutron dose into account. Researchers have relied mainly on measurements and Monte Carlo-based simulation in order to learn more about neutron exposures. However, the time required for these methods is a barrier to research and routine clinical use. Thus, there is a need for fast, accurate analytical models of leakage neutron equivalent dose from proton therapy.

Polf and Newhauser [9] reported that analytical models of neutron equivalent dose from proton therapy are feasible. One proposed analytical model employed a power law to predict neutron equivalent dose per therapeutic dose (H/D) for passively scattered proton therapy for different field sizes and locations within a treatment vault [10,11]. This model was refined for 250 MeV pristine proton beams, both in-air and in a water phantom, by Zhang *et al.* [12]. Anferov [13] reported a model based on shielding calculation methods to predict the equivalent dose from neutrons for 100, 150, and 200 MeV proton beams. The most compete and realistic leakage model to date was reported by Perez-Andujar *et al.* [14], which takes into account four separate neutron energy regimes to predict H/D in-air and in-water for proton beams with energies between 100 and 250 MeV. The model was found to have good agreement when compared with benchmarked Monte Carlo simulations. However, this model was not continuous with proton beam energy and required interpolation of parameters between the discrete energies considered. The model’s large number of parameters made its configuration and use difficult. Furthermore, the model was not compared with measured data nor was it tested at proton beam energies below 100 MeV.

The purpose of this study was to improve an analytical model of neutron H/D by making it continuous in energy and reducing the number of free parameters, thus simplifying its configuration and use. We compared the results of this model with Monte Carlo simulated neutron H/D values between 100 and 250 MeV for a conventional proton therapy beamline. Additionally, we configured and tested a version of the model with new H/D measurements at 75 MeV proton beam energy.

## 2. Methods

### 2.1. Analytical Model

Building upon the methods of Perez-Andujar *et al.* [14], we improved an analytical model for H/D from leakage neutrons from passively scattered proton therapy. For the reader’s convenience, we briefly review the previous model here. H/D contributions are calculated from four neutron energy regimes: intranuclear cascade neutrons (also called direct neutrons), evaporation neutrons, epithermal neutrons (also called 1/E neutrons), and thermal neutrons. Cascade neutrons are produced when a bombarding proton interacts with a target nucleus and can have energies up to the maximum energy of the proton beam. The second highest energy regime, that of evaporation neutrons, corresponds to neutrons ejected by the excited nucleus after the initial proton collision in processes known as compound emission and pre-equilibrium emission. The third energy regime, epithermal neutrons, corresponds to neutrons that have lost some portion of their energy via inelastic scattering and moderation. Some of these neutrons will be lost via capture processes. Finally, the lowest energy regime corresponds to thermal neutrons that have lost most of their kinetic energy and are in thermal equilibrium with the environment. These undergo elastic scattering until they are eventually captured.

**Figure 1:**
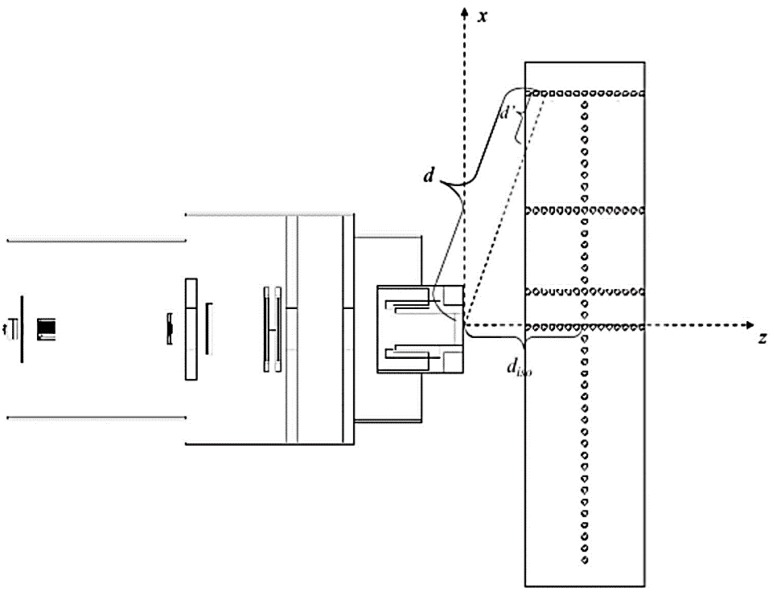
Geometry of a general-purpose proton treatment head and water phantom. Adapted from Perez-Andujar *et al.* [14].

The analytical model for H/D at a point, p, in a water phantom is

(1) $${\left(\frac{H}{D}\right)}_{p}={\left(\frac{H}{D}\right)}_{E,iso}{\left(\frac{d}{d_{iso}}\right)}^{-q}\overset{4}{\underset{i=1}{\sum }}C_{i}\left(E\right)\exp\left[-\alpha_{i}\left(d^{\prime }-d_{iso}^{\prime }\right)\right]\exp\left[\frac{-\left(x^{2}+y^{2}\right)d_{iso}^{2}}{2\sigma_{i}^{2}z^{2}}\right]$$

where ${\left(H/D\right)}_{E,iso}$ is the total neutron equivalent dose per treatment dose at isocenter as a function of the proton beam energy; d is the distance from the neutron source to the calculation point; $d^{\prime }$ is the distance along the ray, d, from the phantom surface to the calculation point; $d_{iso}$ is the distance from the neutron source to isocenter; and $d_{iso}^{\prime }$ is the distance along the ray, $d_{iso}$, from the phantom surface to isocenter. The irradiation geometry, dimensions, and distances are shown in Figure 1. The exponent, q, governs the power law falloff of neutron dose with distance from isocenter. The $C_{i}\left(E\right)$ terms apportion the fraction of the total equivalent dose resulting from the each of the four neutron energy regimes. The first exponential term models neutron attenuation in the phantom. The mean free paths of the neutrons of the $i^{th}$ regime in water are denoted by $\alpha_{i}$, with the first exponential term modeling neutron attenuation in the phantom. The second exponential term, then, models the lateral distribution of the $i^{th}$ neutron regime with $\sigma_{i}$ as the Gaussian width parameter.

In this work, several improvements have been made to the model. Previously, the model required interpolation of ${\left(H/D\right)}_{iso}$ values at proton beam energies between energies contained in the lookup tables. In this work, ${\left(H/D\right)}_{E,iso}$ has been parameterized with proton beam energy according to a power law relationship, or

(2) $${\left(\frac{H}{D}\right)}_{E,iso}=\alpha_{E}\times E^{p_{E}}\times {\left(\frac{H}{D}\right)}_{ref,iso}$$

where ${\left(H/D\right)}_{ref,iso}$ is the neutron equivalent dose value at isocenter for a proton beam of a given reference energy (100 MeV in this study), $p_{E}$ is the exponent governing the power law, $\alpha_{E}$ is a scaling factor, and E is the proton beam energy. This value may be obtained from measurement or from a Monte Carlo simulation and is found by taking the quotient

(3) $${\left(\frac{H}{D}\right)}_{ref,iso}=\frac{{\left(H/p\right)}_{ref,iso}^{closed}}{{\left(D/p\right)}_{ref,iso}^{open}}\;$$

where ${\left(D/p\right)}_{ref,iso}^{open}$ is the absorbed dose in gray per proton at isocenter found with the collimator open and ${\left(H/p\right)}_{ref,iso}^{closed}$ is the neutron equivalent dose in sievert per proton with the collimator closed [11].

The previous model relied on lookup tables of 32 $C_{i}$ values that were found from an iterative fitting process; four values at each of the eight proton beam energies considered from 100 to 250 MeV [14]. In the improved model, the $C_{i}\left(E\right)$ values that apportion the contributions from each neutron regime were parameterized as functions of proton beam energy. Specifically, for intranuclear cascade neutrons, we use the linear form

(4) $$C_{1}\left(E\right)=a_{1}E+b_{1}\;$$

where E is the proton beam energy, $a_{1}$ is the slope, and $b_{1}$ is the intercept. For evaporation neutrons, the cumulative normal was used with a lower bound as in,

(5) $$C_{2}\left(E\right)=a_{2}\text{cnorm}\left(E,b_{2},c_{2}\right)+d_{2}\;$$

where $a_{2}$ is a scaling coefficient, $d_{2}$ is the lower bound, and the cumulative normal function with mean value $b_{2}$ and width parameter $c_{2}$ is defined in the usual way as

(6) $$\text{cnorm}\left(E,b_{2},c_{2}\right)=\frac{1}{c_{2}\sqrt{2\pi }}\int_{-\infty }^{E}\exp\left[\frac{-{\left(E^{\prime }-b_{2}\right)}^{2}}{2c_{2}^{2}}\right]dE\prime \;$$

The epithermal regime was modeled as

(7) $$C_{3}\left(E\right)=a_{3}\;$$

where $a_{3}$ is a constant. For the thermal neutrons, we used

(8) $$C_{4}\left(E\right)=a_{4}E^{2}+b_{4}E+c_{4}\;$$

where $a_{4}$, $b_{4}$, and $c_{4}$ are the polynomial’s second, first, and zeroth order coefficients. Since these curves are used to apportion the equivalent dose from each neutron energy regime, they were constrained so that their sum is unity. The forms of Equations (4)–(8) were chosen empirically to faithfully reproduce the shapes of the C curves with energy while simultaneously reducing the number of model parameters. Specifically, the approach of Perez-Andujar *et al.* [14] required a lookup table containing 40 values plus 9 energy independent parameters for a total of 49 parameters. Our model requires only 13 values plus 9 energy independent parameters for a total of 22 parameters to cover the same interval of proton beam energies from 100 to 250 MeV. Parameterizing these terms with energy offers several advantages compared with the table lookup. It allows the model to be continuous in energy and reduces the number of free parameters. The values for the parameters $\alpha_{E},$ $p_{E},$ $a_{1},$ $b_{1},$ $a_{2},$ $b_{2},$ $c_{2},$ $d_{2},$ $a_{3},$ $a_{4},$ $b_{4},$ and $c_{4}$ were obtained via the iterative fitting process described in Section 2.4 below.

### 2.2. Monte Carlo Simulated H/D Values for General Purpose Beamline at 100 to 250 MeV

Previous studies [12,14] utilized dosimetric data exclusively from Monte Carlo simulations to develop the model. In this study, we purposefully utilized the same Monte Carlo data in order to facilitate the comparison of results with and without the improvements developed in this work. The Monte Carlo data were taken from a simulation of the passive scattering system in place at The University of Texas MD Anderson Cancer Center described in detail by Perez-Andujar *et al.* [14]. This was accomplished with the Monte Carlo Proton Radiotherapy Treatment Planning (MCPRTP) system [15] which utilizes the Monte Carlo N-Particle eXtended (MCNPX) Radiation Transport Code [16]. MCNPX is commonly used for simulating neutron exposures and has been extensively benchmarked against measurements [17,18,19,20,21,22]. Simulations were carried out first with an open collimator to determine the primary absorbed dose per proton, D/p. Next, simulations were done with a closed final collimator to determine the neutron equivalent dose per proton, H/p, and the ratio of these yields H/D. The simulated neutron data includes nominal proton beam energies of 100, 120, 140, 160, 180, 200, 225, and 250 MeV with a closed collimator, pristine Bragg peak, and with the proton beam incident on a water phantom. The phantom contained 100 spherical detecting volumes, each of 1 cm diameter. The detecting volumes were located along lines parallel to the beam axis at 0 cm, 10 cm, 40 cm, and 80 cm off-axis, as well as one line perpendicular to the beam axis at the depth of isocenter in the phantom (22 cm). The simulation geometry is illustrated in Figure 1.

### 2.3. Measured H/D for Ocular Beamline at 75 MeV

Measurements of H/D for the 75 MeV proton beam were carried out at *Centre de Proton thérapie d’Orsay* (CPO) in France in a single-scattering proton beamline dedicated to ocular tumor treatments. During the neutron measurements, a closed patient collimator was used together with a pristine Bragg peak. We selected 75 MeV proton beam energy because it is representative of ocular treatments at CPO. Measurements were taken in air.

Two instrument types were used to acquire neutron ambient dose equivalent, $H^{*}\left(10\right)$, in air. The Berthold LB 6411 [23] is a conventional neutron probe with a spherical polyethylene moderator (25 cm external diameter) and a central ^3^He proportional counter (4 cm external diameter and 10 cm length). It is known to be suitable for ambient dose equivalent measurements in the energy range from thermal to 20 MeV [24]. Additionally, this rem-counter is characterized by a high rejection coefficient for gamma radiation. The WENDI-II is a survey meter with a cylindrical polyethylene moderator (22.9 cm in diameter and 21 cm long), and a central cylindrical ^3^He proportional counter [25]. The moderator encloses a tungsten powder shell of 1.5 cm thickness, which enhances the accuracy of the instrument’s response at energies above about 20 MeV by neutron multiplication.

**Figure 2:**
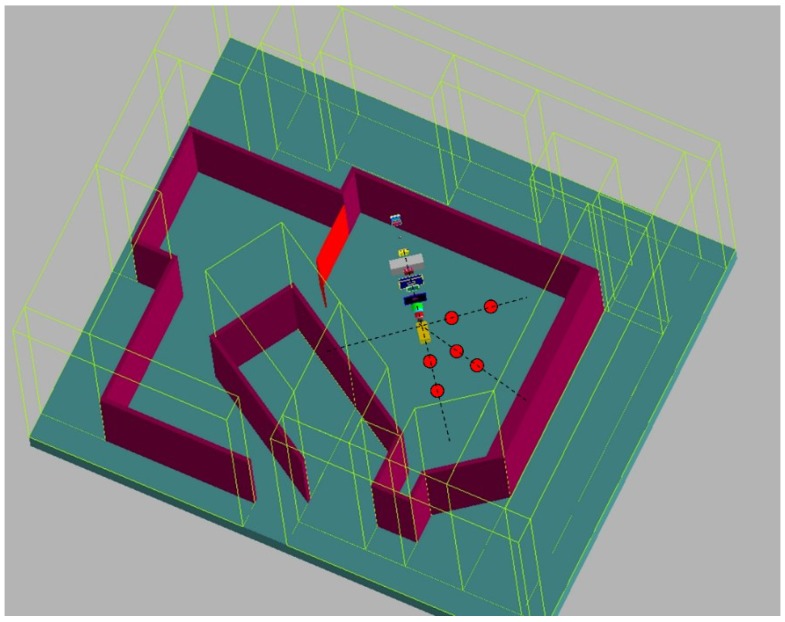
Geometry of a proton therapy system for ocular treatments and positioning of the neutron equivalent dose meters along the three axes at 0°, 45° and 90° with respect to the proton beam direction.

The measurements of $H^{*}\left(10\right)$ were made at isocenter and several distances from isocenter along the proton beam axis and 45° and 90° with respect to the beam axis. Figure 2 shows the ocular beamline and the 10 measurement positions.

### 2.4. Model Training

Previously, the model was trained separately at each proton beam energy considered [14]. In this work, we trained the improved model for the general-purpose beamline by fitting to all data from 100 to 250 MeV simultaneously. All free parameters were selected using the generalized reduced gradient method to minimize the local relative differences in H/D [26].

The model was trained separately for the 75 MeV measurements. This was necessary because of the considerable differences between the beamlines. Because the measured data from the ocular beamline consist of a single proton beam energy and measurements were taken in air, some modifications were necessary. The $\alpha_{i}$ terms from Equation (1), which model neutron attenuation in water, were defined to be zero since there is no water present. The power law model for ${\left(H/D\right)}_{E,iso}$ in Equation (2) was simply replaced with the measured H/D value at isocenter at 75 MeV. Finally, the $C_{i}\left(E\right)$ curves defined in Equations (4)–(8) were replaced with scalar coefficients $C_{1},\;C_{2},\;C_{3},$ and $C_{4}$.

## 3. Results

### 3.1. Model Agreement with Monte Carlo Data at 100 to 250 MeV

**Figure 3:**
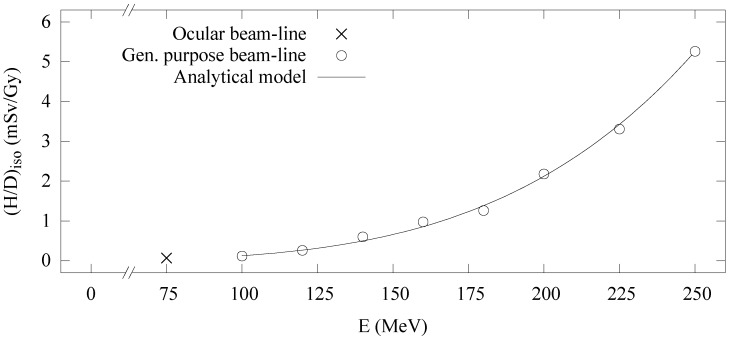
${\left(H/D\right)}_{iso}$ values *versus* proton beam energy, E. Ocular beamline value (×) was measured in-air. General-purpose beamline values (circles) were obtained from Monte Carlo simulations. Analytical model (line) values were calculated using Equation (2).

**Figure 4:**
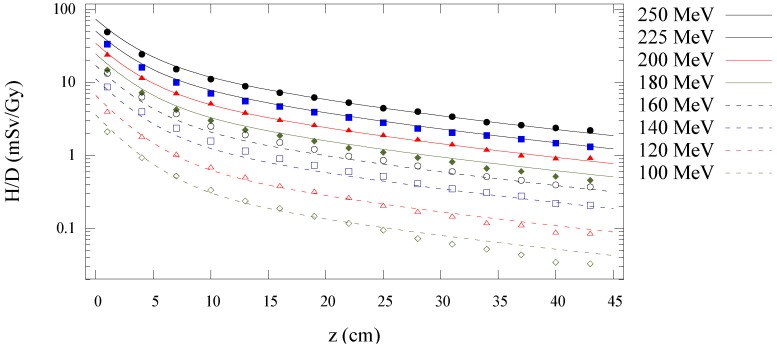
Predictions from Monte Carlo (points) and analytical model (lines) of neutron equivalent dose per treatment dose (H/D) values *versus* depth in water from proton beams for 100, 120, 140, 160, 180, 200, 225, and 250 MeV.

**Figure 5:**
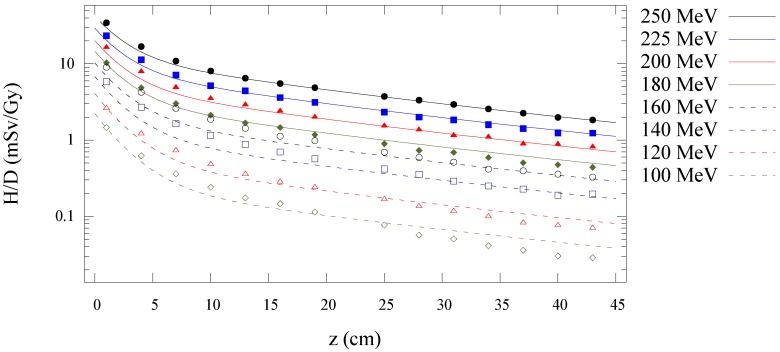
Predictions from Monte Carlo (points) and analytical model (lines) of neutron equivalent dose per treatment dose (H/D) *versus* depth in water at 10 cm off-axis for 100 to 250 MeV proton beam energies.

**Figure 6:**
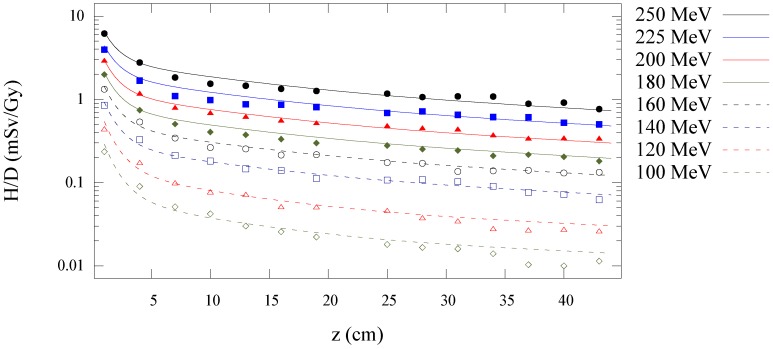
Predictions from Monte Carlo (points) and analytical model (lines) of neutron equivalent dose per treatment dose (H/D) *versus* depth in water at 40 cm off-axis for 100 to 250 MeV proton beam energies.

**Figure 7:**
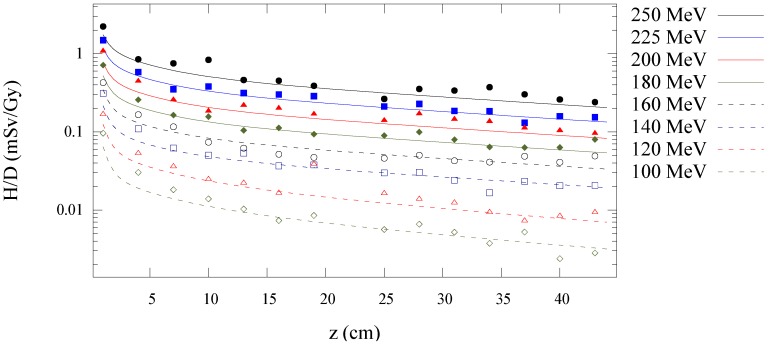
Predictions from Monte Carlo (points) and analytical model (lines) of neutron equivalent dose per treatment dose (H/D) *versus* depth in water at 80 cm off-axis for 100 to 250 MeV proton beam energies.

**Figure 8:**
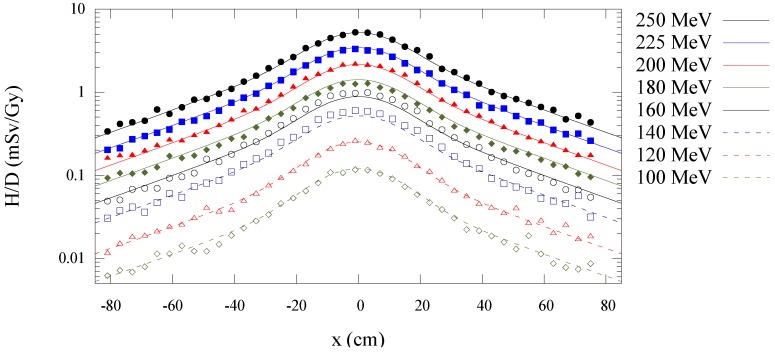
Predictions from Monte Carlo (points) and analytical model (lines) of neutron equivalent dose per treatment dose (H/D) *versus* off-axis position at 22 cm depth in water for 100 to 250 MeV proton beam energies.

Figure 3 shows the Monte Carlo simulated and analytical model calculated values of H/D at isocenter for the general-purpose beamline. H/D along the central axis is plotted as a function of depth in water for all energies in Figure 4. Figure 5, Figure 6 and Figure 7 plot the corresponding results at off-axis distances of 10 cm, 40 cm, and 80 cm, respectively. Figure 8 shows lateral H/D profiles for all energies at isocenter depth (22 cm). These figures demonstrate the good agreement between the H/D values from Monte Carlo simulations and the analytical model calculations. The average relative difference between the analytical model and the Monte Carlo calculations at all proton beam energies and locations considered was 10% with a maximum difference of 60%. The maximum difference occurred for the 120 MeV proton beam energy at a location 80 cm off-axis and 19 cm deep in the phantom.

**Figure 9:**
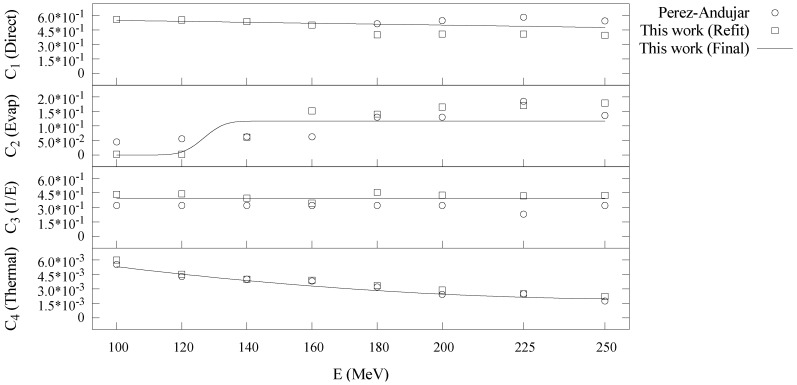
Plot of $C_{i}$ values for each neutron energy regime *versus* proton beam energy. Points represent $C_{i}$ values from Perez-Andujar *et al.* [14] (circles) and from a refitting of that model using the improved fitting methods (squares). The solid curves represent the parameterized $C_{i}\left(E\right)$ models used in the final version.

The parameters governing ${\left(H/D\right)}_{E,iso}$ from (2) are shown in Table 1. Table 2 lists the parameters that govern the $C_{i}\left(E\right)$ curves in Equations (4)–(7). Our results confirm the findings of Perez-Andujar *et al.* [14] that the largest contribution to H/D is from the high-energy direct neutrons followed by the epithermal neutrons. H/D from the evaporation neutron regime is more prevalent at higher energies, and the thermal neutron regime contributes a relatively small component of the equivalent dose. Figure 9 shows the $C_{i}\left(E\right)$ curves from this work plotted with proton beam energy and compared with analogous values from Perez-Andujar *et al.* [14]. Use of the parameterized $C_{i}\left(E\right)$ curves greatly reduced the difficulty of configuring the model by preventing non-physical fluctuations of several parameters with energy, eliminating the need for subjective manual adjustments.

**Table 1:** Parameters for power law relationship for ${\left(H/D\right)}_{E,iso}$.

$\alpha_{E}$	$p_{E}$	${\left(H/D\right)}_{100,iso}$ (Sv/Gy)
8.0 × 10^−9^	4.1 × 10^0^	1.2 × 10^−4^

**Table 2:** Parameters for $C_{i}\left(E\right)$ equations to apportion equivalent dose from neutron energy regimes.

Neutron Energy Regime	$a_{i}$	$b_{i}$	$c_{i}$	$d_{i}$
Intranuclear Cascade	−4.8 × 10^−4^	6.0 × 10^−1^	N/A	N/A
Evaporation	1.2 × 10^−1^	1.3 × 10^2^	5.0 × 10^0^	−1.3 × 10^−11^
Epithermal	4.0 × 10^−1^	N/A	N/A	N/A
Thermal	1.2 × 10^−7^	−6.6 × 10^−5^	1.1 × 10^−2^	N/A

Table 3 lists the energy independent parameters, including the neutron attenuation factors and Gaussian width parameters. As expected, the width parameters for the low energy neutron regimes, epithermal and thermal, are very large corresponding to an isotropic distribution. The higher energy neutron regimes are forward peaked. The exponent governing falloff, q, was found to be 1.13. Table 4 lists a comparison of the accuracies for all locations and all energies, as well as at each specific energy, between this work and Perez-Andujar *et al.* [14]. We found similar agreement with the Monte Carlo simulations. The average local relative differences were within 4% of each other for all energies. The maximum local relative error was decreased from 76% to 60%.

**Table 3:** Neutron attenuation parameters and Gaussian width parameters for the four neutron regimes.

Direct		Evaporation		Epithermal		Thermal	
$\alpha_{1}$ (cm^−1^)	$\sigma_{1}$ (cm)	$\alpha_{2}$ (cm^−1^)	$\sigma_{2}$ (cm)	$\alpha_{3}$ (cm^−1^)	$\sigma_{3}$ (cm)	$\alpha_{4}$ (cm^−1^)	$\sigma_{4}$ (cm)
1.3 × 10^−2^	1.4 × 10^1^	1.3 × 10^−2^	7.7 × 10^1^	3.2 × 10^−2^	3.9 × 10^3^	3.3 × 10^−1^	3.9 × 10^3^

### 3.2. Model Agreement with Measured Data at 75 MeV

**Figure 10:**
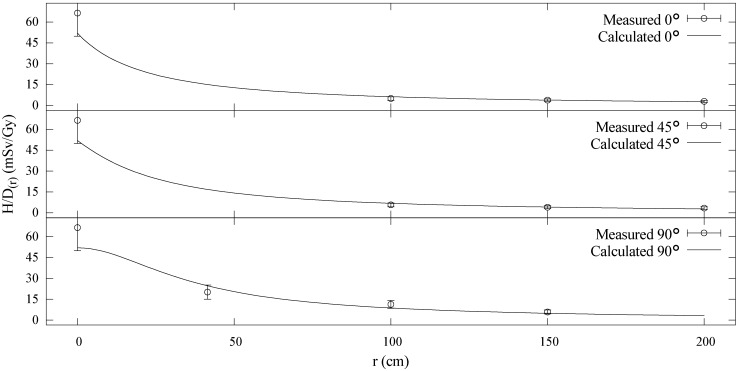
Measured (circles) and calculated (lines) H/D values for the ocular beamline plotted *vs.* distance from isocenter. The top plot shows data taken along the beam axis with points distal to isocenter. The middle plot shows the values along a ray 45° with respect to the beam axis. The lower plot shows values directly lateral to isocenter, *i.e.*, along a ray 90° with respect to the beam axis.

Measured and calculated H/D values for the ocular beamline data are shown in Figure 10 with separate plots for each of the rays measured along: 0°, 45°, and 90° with respect to the beam axis. The plot shows good agreement between measured values and the analytical model. We conservatively estimated the uncertainty in the measured data at ±25%, and the calculated H/D values agreed within this limit for all points considered. The average error was 16% and the maximum error was 24%. The exponent that governs falloff, q, was found to be 1.5. The larger q value is expected in this instance since the ocular beamline is narrower and should more closely resemble a point source. The parameters $C_{1}-C_{4},\;\sigma_{1}-\sigma_{4},$ and $H/D_{iso}$ for the ocular beamline data are listed in Table 5.

**Table 4:** Average local relative error ($\bar{\Delta }$) and maximum local relative error ($\Delta {|}_{\text{max}}$) for the analytical model of the general-purpose beamline from this work and Perez-Andujar *et al.* [14].

Proton Energy (MeV)	This Work		Perez-Andujar *et al.* [14]	
	$\bar{\Delta }\;\left(\%\right)$	$\Delta {|}_{max}\;\left(\%\right)$	$\bar{\Delta }\;\left(\%\right)$	$\Delta {|}_{max}\;\left(\%\right)$
All	10	60	10	76
250	7	39	7	30
225	7	29	6	29
200	7	31	6	34
180	11	31	7	34
160	11	33	10	45
140	11	45	10	46
120	11	60	15	61
100	16	54	18	76

**Table 5:** Model parameters for ocular beamline.

$C_{1}$	$C_{2}$	$C_{3}$	$C_{4}$	$\;\sigma_{1}\;\left(cm\right)$	$\;\sigma_{2}\;\left(cm\right)$	$\;\sigma_{3}\;\left(cm\right)$	$\;\sigma_{4}\;\left(cm\right)$	${\left(H/D\right)}_{iso}\;\left(Sv/Gy\right)$
1.1 × 10^−2^	7.8 × 10^−1^	1.0 × 10^−1^	9.6 × 10^−2^	9.4 × 10^0^	1.0 × 10^3^	4.2 × 10^3^	4.2 × 10^3^	5.2 × 10^−5^

## 4. Discussion

We have improved an analytical model for predicting H/D from leakage neutrons for proton therapy by parameterization of energy dependent aspects of the model, thereby reducing the number of free parameters and simplifying the model configuration process. We have demonstrated the training of this model using Monte Carlo simulated neutron exposures for proton beam energies from 100 to 250 MeV and using measured data from a separate 75 MeV proton therapy beamline.

The major finding of this work is that an analytical model of neutron H/D for passively scattered proton therapy may be applied continuously over a wide range of proton beam energies with relatively fewer model parameters. In addition to reducing the number of free parameters, explicitly modeling the contribution of different neutron energy regimes based on the proton beam energy yields other advantages to our model. The model is now continuous in energy, and this approach obviates the need to interpolate from a table of values at intermediate energies. The model can be easily applied to energies between those used in the work without the need for additional measurements or crude linear interpolation. In contrast, the lookup table approach employed by Perez-Andujar *et al.* [14] requires the interpolation of several parameters for use at energies other than those contained in the lookup table and also requires a large number of energy specific parameters that complicate the training of the model. Our method reduces the number of free parameters, and our improved fitting methods have made the model training process much simpler for the user. This approach gives confidence that the fitted parameters follow physically realistic dependencies on proton beam energy and are not the result of over-fitting or memorizing the data.

Another encouraging confirmatory finding is that the analytical model is applicable to low energy proton beams such as those used in ocular treatments. It is important that the model be easily adaptable to other passive scattering treatment systems, so that it can have the greatest possible impact and find use at many different institutions. Importantly, Farah *et al.* [27] previously reported on the difficulty in configuring the original model, an obstacle that this work has successfully overcome.

The results of this work are consistent with the findings of other studies. Perez-Andujar *et al.* [14] found that to accurately model the equivalent dose from leakage neutrons requires the consideration of no fewer than four neutron energy regimes. That is supported by this work. The contributions from each of the four neutron energy regimes are similar for this work and the previous model. Furthermore, the dosimetric accuracies were found to be similar.

A major strength of this study is that the improved model relies on far fewer free parameters than previous works. The inclusion of measured data from a second passively-scattered proton therapy beamline is another strength of this study. Specifically, the analytical model was configured for use at the lower energy (75 MeV) and compared against experimental data to validate its utility to predict stray radiation from an ocular beamline.

One limitation of this study is that we only benchmarked the model with measured data at a single proton beam energy for the ocular beamline. Additionally, the measured data was taken in-air and not in a water phantom. These limitations are minor because we demonstrated good agreement for the model compared with Monte Carlo simulated H/D values in a water phantom and at many different energies for the general purpose beamline.

Future work on leakage radiation from proton therapy should include research and development to translate the analytical models to clinical practice. Specifically, the model should be integrated into treatment planning systems to facilitate routine clinical dose assessments for patients with heterogeneous anatomy and irregular external surfaces. A study from our group has yielded promising preliminary results indicating that the integration of a similar analytical model into a radiotherapy treatment planning system is technically feasible and the leakage-dose algorithm is sufficiently fast for routine clinical treatment planning applications [28]. Specifically, this study found that the time required was a factor of 1.6 of the time necessary for the proton dose calculation allowing for the total calculation to be completed in less than one hour on a single CPU. Additional research and development work will be needed to enhance the analytical model to account for other treatment factors such as range modulation. Range modulation can be modeled from first principles using the proton modulation function including proton fluence weights, Equations (2)–(5) from Polf and Newhauser [9] or Equations (1)–(4) from Zheng *et al.* [29], and dosimetric data at multiple proton beam energies from Monte Carlo simulations. If the relationship between H/D and the modulation width is known from measurements or simulations of the usual case with flat-topped Bragg peaks, the dependence of H/D on modulation width may be accounted for with an empirical analytical model. From previous work, we know that H/D increases with modulation width modestly and continuously (see Figure 9 in Zheng *et al.* [29]), and it appears to follow a simple analytic expression, e.g., a polynomial or asymptotic exponential function. Studies on range modulation and other treatment factors are underway in our laboratory. This model may also find application for scanned-beam proton therapy beamlines equipped with passive and dynamic collimators, e.g., milled brass collimators, multi-leaf collimators [30,31], and trimmers [32].

## 5. Conclusions

In this work, we improved an analytical model of neutron H/D for passively scattered proton therapy in the energy range from 100 to 250 MeV. The improved model relies on fewer configuration parameters and is easier to train. We tested the analytical model on measured neutron H/D values from a separate 75 MeV beamline. Our results revealed good agreement of the model with both measured data and Monte Carlo simulations. The results of this work suggest that, with further development and testing, analytical models may be applicable for routine use in clinical treatment planning systems to predict neutron exposures to patients.
